# (E)3-2-(1-(2,4-Dihydroxyphenyl)ethyldeneamino)phenyl)-2-methylquinazoline-4(3H)-one Schiff Base and Its Metal Complexes: A New Drug of Choice against Methicillin-Resistant *Staphylococcus aureus*

**DOI:** 10.1155/2014/343540

**Published:** 2014-03-09

**Authors:** K. Siddappa, Sunilkumar B. Mane, Deene Manikprabhu

**Affiliations:** ^1^Department of Post-Graduate Studies and Research in Chemistry, Gulbarga University, Gulbarga, Karnataka 585106, India; ^2^Department of Microbiology, Gulbarga University, Gulbarga, Karnataka 585106, India

## Abstract

The 3-(2-aminophenyl) quinazolin-2-methyl-4(3H)-one and 2,4-dihydroxyacetophenone undergo condensation to afford (E)3-2-(1-(2,4-dihydroxyphenyl)ethyldeneamino)phenyl)-2-methylquinazoline-4(3H)-one Schiff base (DHPEAPMQ). The newly synthesized Schiff base (DHPEAPMQ) and its metal complexes were evaluated for their antimicrobial activity against methicillin-resistant *Staphylococcus aureus* isolated from the Gulbarga region in India. The Cu(II), Ni(II), and Zn(II) complexes of Schiff base (DHPEAPMQ) showed good antimicrobial activity. So, this could be a new drug of choice.

## 1. Introduction 

Bioinorganic chemistry is an emerging interdisciplinary field of science that utilizes Schiff bases and their transition metal complexes for various applications in biological, medical, and environmental sciences [1]. The Schiff base ligands are considered as “privileged ligands” because they are easily prepared by the condensation between aldehydes and imines, and their use of analytical and biological processes on different models has solved many serious problems [2, 3]. Over the past decade, the synthesis of the privileged classes of heterocyclic molecules has become one of the main areas of interest in synthetic chemistry [1, 2]. These important structures have gained much attention, owing to their potential role as ligands, which are capable of binding multiple biological targets [3]. Among nitrogen-containing heterocyclic molecules, substituted quinazolinones and quinazolines are considered as important therapeutic scaffolds [4]. Quinazoline-4(3H)-one and its derivatives gained extensive importance in medicinal chemistry because of their diverse pharmacological activities such as, antibacterial, antifungal, anti-inflammatory, anticancer, anticonvulsant, antioxidant, antitubercular, anti-HIV, and analgesic [5].

*Staphylococcus aureus* (*S. aureus*) resistant to methicillin is a major problem that the world is now facing. The antibiotic era, barely 60 years old, is also threatened because of increase of resistance rhythm of this organism against different antibiotics [6]. Today's challenging task is to synthesize a new antimicrobial agent that does not generate microbial resistant so studies in finding out new antimicrobial agents against methicillin-resistant* Staphylococcus aureus* (MRSA) are desperately required if public health crisis is to be averted. Substitutes for antibiotics are the Schiff base and its metal complexes owing high biological activity. Based on the biological importance of quinazoline Schiff base and to find a new antimicrobial agent against MRSA an effort was made to synthesize a new (E)3-2-(1-(2,4-dihydroxyphenyl)ethyldeneamino)phenyl)-2-methylquinazoline-4(3H)-one (DHPEAPMQ) Schiff base and its complexes. The present investigation deals with the synthesis of Schiff base (DHPEAPMQ) and its complexes for evaluation of antimicrobial activity against MRSA.

## 2. Materials and Methods

All the reagents and chemicals were of AR grade. Elemental analyses (C, H, and N) were carried out on the Perkin Elmer 240C model. IR spectra of the Schiff base (DHPEAPMQ) and its complexes in KBr pellets were recorded using Perkin Elmer Spectrum one FT-IR spectrometer in the spectral range 4000–350 cm^−1^. The electronic spectra of the Cu(II) and Ni(II) complexes were recorded on a ELICO SL-164 double beam UV-Vis spectrophotometer in the range 200–1100 nm using dimethyl formaldehyde(DMF) as a solvent. The ^1^H NMR spectra were recorded on AMX-400 NMR spectrometer, using tetramethylsilane (TMS) as an internal standard and DMSO-d_6_ as a solvent. Mass spectra were recorded on JEOL GCMATE II GC-MS mass spectrometer. Magnetic susceptibilities were measured using a Gouy balance at room temperature using Hg[Co(NCS)_4_] as calibrant. The molar conductance data were recorded on the ELICO-CM-82T conductivity bridge in DMF solution at concentration ~10^−3^ M and EPR spectra recorded on Bruker Biospin.

### 2.1. Synthesis of 3-(2-Aminophenyl)-2-methylquinazolin-4(3H)-one

In 100 mL round bottom flask a homogeneous mixture of 2-methyl-aminobenzoate (0.01 mol) and o-phenylenediamine (0.01 mol) in ethanol (25 mL) was mixed gently and heated to reflux on a water bath for 2-3 h. The resulting mixture obtained after reflux was separated as a solid product. The solid product obtained was filtered, washed with hot ethanol, and finally recrystallized from toluene.

### 2.2. Synthesis of Schiff Base (DHPEAPMQ)

**Scheme 1:**
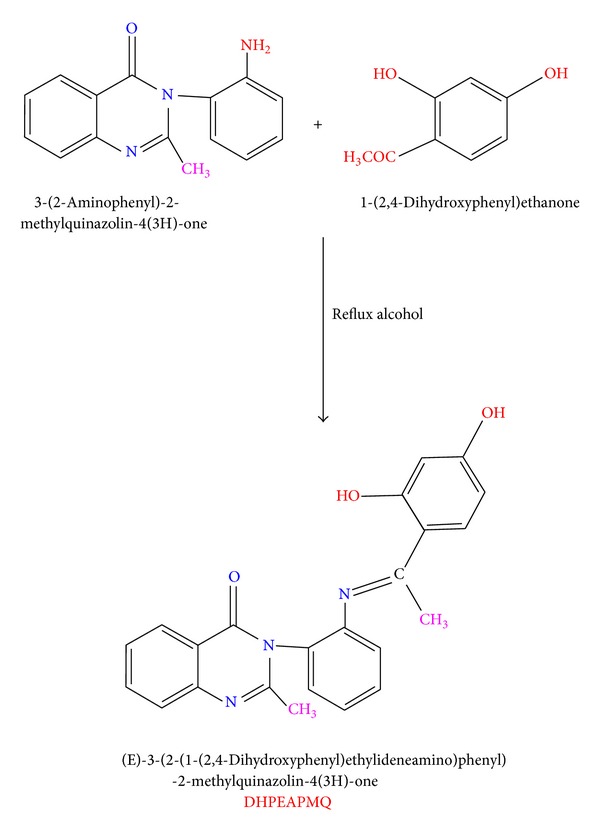
Synthetic route for the preparation of (DHPEAPMQ).

The synthetic pathway involved in the synthesis of Schiff base (DHPEAPMQ) mentioned below: a 30 mL hot alcoholic solution of 3-(2-aminophenyl)-2-methylquinazolin-4(3H)-one (0.01 mol) and 20 mL of 1-(2,4-dihydroxyphenyl) ethanone (0.01 mol) was refluxed for about 4-5 h on water bath. On evaporating the solvent the solid product was separated out, filtered, followed by washing with ethanol, and finally recrystallized from hot ethanol to give (DHPEAPMQ) as shown in Scheme 1.

### 2.3. Synthesis of Metal Complexes

For the preparation of representative Cu(II), Ni(II), and Zn(II) complexes, a solution (30 mL) of the Schiff base (DHPEAPMQ) in hot methanol was added to a stirred solution of metal(II) chloride in 20 mL methanol. The mixture was refluxed for 3 h at a temperature of ~78°C. The reaction mixture maintained to pH 6.0–7.0 using sodium acetate, and solid intense coloured complexes formed were precipitated out. The precipitated complexes were further refluxed for about an hour to check their stability. Later they were filtered off, washed thoroughly with water and little warm methanol for apparent dryness, and finally dried in a vacuum desiccator fused over CaCl_2_.

### 2.4. Isolation and Identification of MRSA

Samples like blood, pus, and other exudates were obtained from different hospitals and health care centers of the Gulbarga region in India. All the samples were first inoculated onto blood agar (Hi-media) plates. The plates were incubated at 37°C for 24–48 h. The colonies obtained on blood agar after incubation were again inoculated onto mannitol salt agar; the plates were again incubated at 37°C for 24–48 h. The preliminary identification of* S. aureus* isolates was detected by change in color of the medium from red to yellow due to mannitol fermentation. Further, the* S. aureus* were identified based on morphological, microscopic, and biochemical tests [6]; among the identified* S. aureus* the MRSA were detected phenotypically using antibiotic susceptibility test as per the guidelines recommended by the Clinical and Laboratory Standards Institute (CLSI-2012) [7].

### 2.5. Antimicrobial Activity of Schiff Base (DHPEAPMQ) and Its Complexes against MRSA

The antibacterial activities of the newly synthesized Schiff base (DHPEAPMQ) and its metal complexs against MRSA were evaluated on Mueller Hinton agar (MHA) by making a lawn of MRSA (0.5 McFarland) with the help of sterile cotton swabs, and wells of 6 mm diameter were punched carefully using a cork borer. The wells were loaded with 100 *μ*L (1 mg/mL in DMSO as a solvent) of different investigated test compounds. The plates were incubated at 37°C for 24 h. Antibacterial activity was determined by measuring the zone of inhibition.

### 2.6. Determination of Minimum Inhibitory Concentration (MIC)

The MIC is that last tube in which no visible growth of microorganism was recorded. To determine MIC different volumes of investigating test compounds (2, 4, 6, 8, 10, 12, 14, 16, 18, 20, 22, 24, 26, 28, 30, 32, 34, 36, 38, and 40 *μ*g/mL) and MRSA culture (0.5 McFarland) were added into Mueller Hinton Broth (MHB) and were incubated at 37°C for 18 h. For comparison purpose methicillin antibiotic was taken as the standard.

## 3. Results and Discussion

MRSA was isolated at low levels a decade ago but is currently widespread [8] due to the development of resistance to methicillin antibiotic which exponentially increased high morbidity and mortality [6, 9] so there is an urgent need of a new antimicrobial agent who does not generate resistance against MRSA. Alternate to antibiotics are Schiff base and their metal complexes (especially quinazoline-4(3H)-one and their metal complexes) having a vast application in the field of medical microbiology. Recently, Prashanth and Revanasiddappa showed antimicrobial activity of glutamine linked 2, 3 disubstituted quinazolinone derivatives as potent antimicrobial agent against both gram positive and gram negative bacteria [10]. Similarly, Kalagouda et al. reported antibacterial activity of Lanthanide(III) Complexes of 2-[2-hydroxy-3-methoxyphenyl]-3-[2-hydroxy-3 methoxybenzylamino]-1,2-dihydroquinazolin-4(3H)-one against bacteria like* Pseudomonas aeruginosa* and* Bacillus cirroflagellosus* [11]. Due to the diverse antimicrobial activity of the Schiff base, in the present investigation we report the synthesis and characterization of new Schiff base (DHPEAPMQ) and its antimicrobial activity against MRSA isolated from the Gulbarga region in India.

### 3.1. Structural Analysis of the Complexes

The analytical and physical parameters of the newly synthesized Schiff base (DHPEAPMQ) and its metal complexes reveal Schiff base (DHPEAPMQ) and its complexes were very stable and nonhygroscopic at room temperature, and the complexes were sparingly soluble in common organic solvents and completely soluble in DMF and DMSO. The measured molar conductance values were in the range of 12–16 Ohm^−1^ cm^2^ mol^−1  ^ which indicates the nonelectrolytic nature of metal complexes [12] as shown in Table 1. The metals and chloride contents were determined as per standard protocol [13].

The formations of prescribed geometries of metal complexes were achieved by various spectral studies such as UV-Visible, IR, ^1^H NMR, and mass.

The electronic spectra of a Schiff base (DHPEAPMQ) exhibit two bands between 33768 and 27397 cm^−1^ due to  *π* → π* and  *n* → π* transitions associated with -C=N and C=O, respectively. The shift in frequencies to higher wavelength suggests the coordination of azomethine nitrogen and carboxylic oxygen with metal ions. The electronic spectrum of Cu(II) complex displayed a broad asymmetric band in the region of 12540–17860 cm^−1^ due to ^2^E_*g*_ → ^2^T_2*g*_ transition, indicating the distorted octahedral geometry around the Cu(II) ion [14]. The Ni(II) complex shows three well-resolved bands at 9382, 18920, and 20860 cm^−1^ assigned to ^3^A_2*g*_(F)→^3^T_2*g*_ (F) (*ν*_1_), ^3^A_2*g*_ (F)→^3^T_1*g*_ (F) (*ν*_2_), and ^3^A_2*g*_ (F)→^3^T_1*g*_ (P) (*ν*_3_) transitions, respectively, which show the octahedral geometry around the Ni(II) ion [15]. The electronic parameter values such as the Racah interelectronic repulsion parameter (*B*′), ligand field splitting energy (10Dq), covalency factor (*β*), and ligand field stabilization energy (LFSE) [16] have been calculated by using band-fitting equation [17]. The *B*′values for the complexes were lower than the free ion values which indicate orbital overlaps and delocalization of d-orbital's. The covalent factor *β* equal to *B*/*B*′ for the complexes was less than one suggesting the considerable covalent character of metal-ligand bonds. In the present study the *β* (0.76) values obtained were less than unity, which indicates the covalency for the metal-ligand bonds. In addition, the covalence factors (b^1/2^), Sinha parameter (*δ*%), that is, metal-ligand covalency percent, and the covalency angular overlap parameter (*η*) have been calculated from the values of *β* by using the following expressions [18]:

(1) $$\begin{array}{c} {\text{b}}^{1/2}\;=\;\frac{1}{2}\left[{\left(1-\beta \right)}^{1/2}\right],\;\;\delta \;\left(\%\right)\;=\;\left[\frac{1-\beta }{\beta }\right]\;\times 100,\\ \eta \;=\;\frac{\left[\left(1-\beta^{1/2}\right)\right]}{\beta^{1/2}}. \end{array}$$

The electronic spectral studies of the Cu(II) and Ni(II) complexes yield a positive value for (1-*β*), b^1/2^, and *δ*% as well as *η* which suggest that the bonding between metal and ligand was covalent in the complexes. The values of the parameter of bonding (*β* ^1/2^) and angular overlap parameter (**η**) were found to be positive which indicates a strong covalent bonding between the Schiff base (DHPEAPMQ) and its complexes as shown in Table 2.

The IR spectrum of Schiff base (DHPEAPMQ) displays a broadband in the region of 3402–3380 cm^−1^ due to *ν*(2-OH) of 1-(2,4-dihydroxyphenyl) ethanone; upon metal complexes formation the disappearance of one (-OH) groups indicates the involvement of phenolic oxygen bonding with metal ion via deprotonation [19]. However, the peak in the region of 3390–3395 cm^−1^ that was retained in complexes shows the presence of uncoordinated (-OH) group. Furthermore, the evidence for the coordination through only one or both of the phenolic oxygen was confirmed by ^1^H NMR spectral studies. The Schiff base (DHPEAPMQ) shows characteristic resonance signals at *δ* 11.43 ppm (s, 1H, OH) and *δ* 9.60 ppm (s, 1H, OH) upon metal complexes formation, disappearance of one -OH proton *δ* (9.60 ppm) on the other hand, the other proton remained unaltered at *δ* 11.43 ppm (s, 1H, OH) (D_2_O exchangeable) indicates the participation of only one phenolic oxygen in coordination with metal ion, via deprotonation [20] as presented in (see supplementary file 1(a–d) in Supplementary Material available online at http://dx.doi.org/10.1155/2014/343540).

A characteristic high intense band due to azomethine *ν*(-C=N) in the IR spectrum of Schiff base (DHPEAPMQ) appeared in the region of 1598–1592 cm^−1^, experiences a negative shift of 15–20 cm^−1^ in their respective complexes, and lower value of *ν*(-C=N) stretching can be explained on the basis of a drift of the lone-pair density of azomethine nitrogen towards the metal ions, indicates coordination of azomethine nitrogen with the metal ions [21]. Further, this was confirmed by NMR spectra, owing to the downfield shift of azomethine proton from *δ* 8.35 ppm (s, 1H, -CH=N) of Schiff base (DHPEAPMQ) to *δ* 8.42 ppm (s, 1H, -CH=N) in complexes, which shows the involvement of -CH=N nitrogen in coordination.

In the IR spectrum of Schiff base (DHPEAPMQ), a high intense strong band in the region of 1712–1702 cm^−1^ was assigned due to the carboxyl group of quinazoline ring (C=O), which shows a downfield shift of 20–30 cm^−1^and indicates the participation of carboxylic oxygen upon complex formation [22].

All the complexes show medium intensity bands in the region of 558–547 cm^−1^ and 463–457 cm^−1^ assigned to *ν*(M-O) and *ν*(M-N) vibrations, respectively, which further support the coordination of the Schiff base (DHPEAPMQ) through the nitrogen of azomethine and carboxylic and phenolic oxygen with various metal ions [23]. Moreover, a weak band was observed in the region of 355–350 cm^−1^ assigned to *ν*(M-Cl); this was a characteristic of the chloride atom in Zn(II) complex and was further confirmed by quantitative chloride estimation. The ^1^H NMR signal corresponds to the rest of the protons such as methyl protons *δ* 2.52–2.64 ppm (s, 6H, and CH_3_) and aromatic protons *δ* 6.71–8.72 ppm (m, 11H, and Ar-H) of Schiff base (DHPEAPMQ) and its complexes were exhibited in their expected regions.

**Figure 1:**
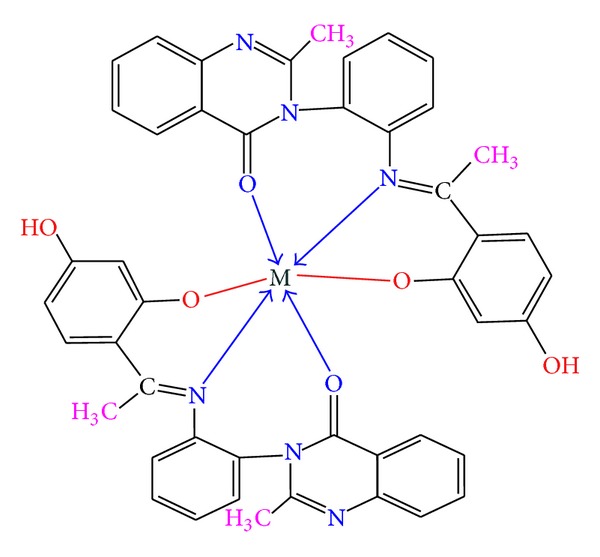
Proposed structures of Cu(II) and Ni(II).

**Figure 2:**
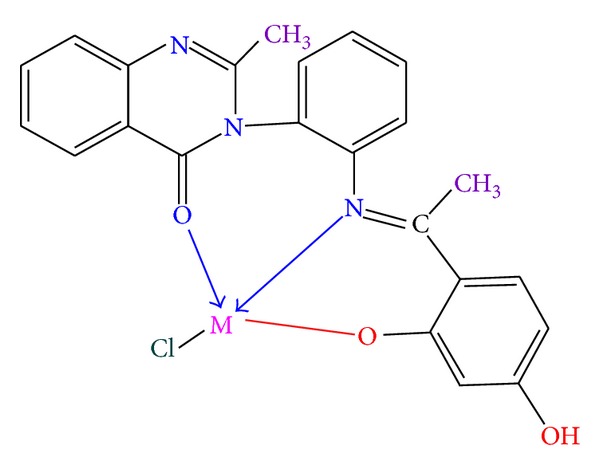
Zn(II) complexes.

Thus from the above, it was inferred that the Schiff base (DHPEAPMQ) acts as a tridentate ONO donor and forms octahedral geometry with Cu(II), Ni(II) complexes, and tetrahedral geometry with Zn(II) complex via the involvement of phenolic oxygen, azomethine nitrogen, and carboxylic oxygen as shown in Figures 1 and 2.

The effective magnetic moments, *μ*_eff_, expressed in multiples of the Bohr Magneton calculated for Cu(II) and Ni(II) complexes were in the range of 1.81–1.96 BM and 3.34–3.69 BM, respectively, due to mononuclear Cu(II) (d^9^, 1 unpaired electron) and Ni(II) (d^8^, 2 unpaired electrons) complexes which indicates their octahedral geometries [24], whereas the Zn(II) complex is diamagnetic in nature.

The formation of a Schiff base (DHPEAPMQ) and its complexes was further confirmed by their mass spectral study. All the spectra exhibit parent peaks due to molecular ions (M^+^) and the isotopic peak owing to the chlorine substitution. The proposed molecular formula of each compound was confirmed by its molecular formula weight with* m/z* values. The mass spectra of Schiff base (DHPEAPMQ) showed the formation of a molecular ion peak at* m/z* 385 [M]^+^, whereas Cu(II), Ni(II), and Zn(II) complexes show the formation of molecular ion peaks along with isotopic peaks at* m/z* 832 [M]^+^, 826 [M]^+^ and 485 [M]^+^, 487 [M+2]^+^, respectively, corresponding to their molecular formula.

#### 3.1.1. ESR Spectrum of Cu(II) Complex

The* X*-band ESR spectra of Cu(II) complex were recorded in the polycrystalline state at room temperature at a frequency of 9.387 GHz with a field set of 3950 G. The information about the hyperfine and super hyperfine structure was obtained, to explain the geometry of the complex as well as the site of the metal-ligand bonding or chemical environment around the metal ion. In the present study the ESR spectral pattern of Cu(II) complex as depicted in supplementary file 1(e) gives the data *g*_||_ = 2.28 and *g*_⊥_ = 2.07, *g*_av_ = 2.11, and  *g*_iso_ = 2.17. The observed *g*_||_ value was less than 2.3 and confirms the strong covalent nature of the metal-ligand bond. The *g*_||_ value plays a significant role in elucidating the metal-ligand bond character, for ionic *g*_||_ > 2.3 and for covalent characters *g*_||_ < 2.3, respectively [25]. The ESR spectrum showed asymmetric bands with *g*_||_ > *g*_⊥_ > 2.0023, representing that the unpaired electrons lay predominantly in the *d*_*x*^2^−*y*^2^_ orbital with possible mixing of *d*_*z*^2^_ because of low symmetry [26]. The* G* value was calculated by using the formula *G* = (*g*_||_ − 2.0023)/(*g*_⊥_ − 2.0023) which was greater than 4 as shown in Table 3 and indicates the negligible exchange interaction in solid complex as suggested by Hathaway and Billing [27].

### 3.2. Antimicrobial Activity of Schiff Base (DHPEAPMQ) and Its Complexes against MRSA

**Figure 3:**
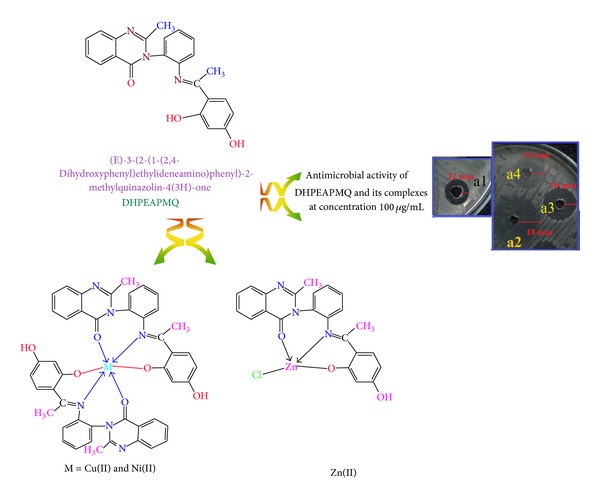
Antimicrobial activity of Schiff base (DHPEAPMQ) and its complexes against MRSA [a1: Schiff base (DHPEAPMQ), a2: Cu(II), a3: Ni(II), and a4: Zn(II) complex].

The antimicrobial activity of the Schiff base (DHPEAPMQ) and its complexes was evaluated against MRSA and isolated from different hospitals and health care centers of the Gulbarga region in India.  Figure 3 shows the good antimicrobial activity of the Schiff base (DHPEAPMQ) against MRSA with a zone of inhibition (12 mm); however, upon complex formation the antimicrobial activity increased with a zone of inhibition (18 mm, 16 mm, and 14 mm) for Cu(II), Ni(II), and Zn(III) complexes, respectively.

The antimicrobial property of the Schiff base was rationalized due to the presence of azomethine (C=N) group; this imports in elucidating the mechanism of transamination and resamination reactions in biological system [28]. The formation of hydrogen bonds through the azomethine group with the active centers of various cellular constituents results in interference with normal cellular processes [29]. Furthermore, it has also been suggested that the Schiff base ligands with nitrogen and oxygen donor systems might inhibit enzyme production causing cell death [30].

The enhanced activity of metal complexes than the Schiff base can be explained by Tweedy's chelation theory, which suggests that the chelation could allow for the delocalization of *π*-electrons over the entire chelate ring and enhances the lipophilicity of the complexes. This increased lipophilicity facilitates the penetration of the complexes into lipid membranes, further restricting the proliferation of the microorganisms [31].

### 3.3. Determination of Minimum Inhibitory Concentration (MIC)

Table 4 shows MIC values of Schiff base (DHPEAPMQ) and its complexes, which indicates Cu(II) as an excellent antimicrobial agent followed by Ni(II) and Zn(III) complexes. The enhanced activity of Cu(II) complex may be due to their particle size and also may be attributed to its higher stability constants [32] when compared to the Schiff base (DHPEAPMQ) and other metal complexes.

## 4. Conclusion

In conclusion, we report the synthesis and characterization of new Schiff base (DHPEAPMQ) and its metal complexes and their antimicrobial activity against MRSA isolated from clinical samples of the Gulbarga region in India. The syntheses were confirmed by UV-visible, IR, NMR, mass, and ESR spectral data and their results reveal that Cu(II) and Ni(II) complexes exhibit an octahedral geometry while Zn(II) complex shows tetrahedral geometry. The MIC values of Cu(II), Ni(II), and Zn(II) were 14, 18, and 24 *μ*g/mL, respectively, which show an excellent antimicrobial activity against MRSA. So the same can be used as a new drug of choice.

## Supplementary Material

The IR and NMR spectra of Schiff base DHPEAPMQ and its metal complex were presented in Supplementary file (1a-d). ESR spectra of Cu(II) complex was given in Supplementary file (1e).

## Figures and Tables

**Table 1:** Elemental analysis of physical and magnetic data of Schiff base (DHPEAPMQ) and its complexes.

Molecular formula of ligand/complexes	Mol. Wt. (g/mol)	m. p. °C	Elemental analysis found (calculated) %					*μ*_eff_ (B.M)	Λ_m_*
			C	H	N	M	Cl		
C_23_H_19_N_3_O_3_ (DHPEAPMQ)	385.42	270	71.17 (71.67)	4.61 (4.97)	10.31 (10.90)	—	—	—	—
[Cu(C_23_H_18_N_3_O_3_)_2_]	832.36	292	66.38 (66.71)	4.01 (4.36)	10.10 (10.45)	7.36 (7.90)	—	1.81	16.30
[Ni(C_23_H_18_N_3_O_3_)_2_]	826.20	285	66.10 (67.77)	4.03 (4.38)	10.01 (10.16)	6.94 (7.09)	—	4.39	12.37
[Zn(C_23_H_18_N_3_O_3_)Cl)]	485.27	291	56.07 (56.93)	3.42 (3.74)	8.52 (8.66)	13.28 (13.48)	7.22 (7.31)	Diam	14.10

*Molar conductance values in Ohm^−1^ cm^2^ mol^−1^.

**Table 2:** Ligand field, Sinha, metal-ligand covalency percent, and covalency angular overlap parameters of Cu(II) and Ni(II) complexes.

Complexes	Dq	B′	B	*β*%	*ν*_2_/*ν*_1_	(1 − *β*)	*b* ^1/2^	*δ*%	*η*	LFSC (Kcal)
[Cu(C_23_H_18_N_3_O_3_)_2_]	1564	—	—	—	—	—	—	—	—	26.81
[Ni(C_23_H_18_N_3_O_3_)_2_]	953	744	0.76	23.3	2.01	0.24	0.24	31.57	0.34	16.33

**Table 3:** ESR data of the [Cu(C_23_H_18_N_3_O_3_)_2_] metal complex.

Complex	*g*_||_	*g*_⊥_	*g*_av_	*g*_iso_	*G*
[Cu(C_23_H_18_N_3_O_3_)_2_]	2.28	2.07	2.11	2.17	4.10

**Table 4:** MIC values of Schiff base (DHPEAPMQ) and its complexes against MRSA.

Compounds	MIC (*μ*g/mL)
Schiff base (DHPEAPMQ)	28
[Cu(C_23_H_18_N_3_O_3_)_2_]	14
[Ni(C_23_H_18_N_3_O_3_)_2_]	18
[Zn(C_23_H_18_N_3_O_3_)Cl]	24
Methicillin	16
