# Supplementary material for: (E)3-2-(1-(2,4-Dihydroxyphenyl)ethyldeneamino)phenyl)-2-methylquinazoline-4(3H)-one Schiff Base and Its Metal Complexes: A New Drug of Choice against Methicillin-Resistant Staphylococcus aureus
Source: Bioinorg Chem Appl. 2014 Mar 9;2014:343540. doi: 10.1155/2014/343540 (PMC3966480; doi:10.1155/2014/343540)
Supplement: Supplementary file 1 — The IR and NMR spectra of Schiff base DHPEAPMQ and its metal complex were presented in Supplementary file (1a-d). ESR spectra of Cu(II) complex was given in Supplementary file (1e). [file 343540.f1.pdf]

Supplimentary file 1.

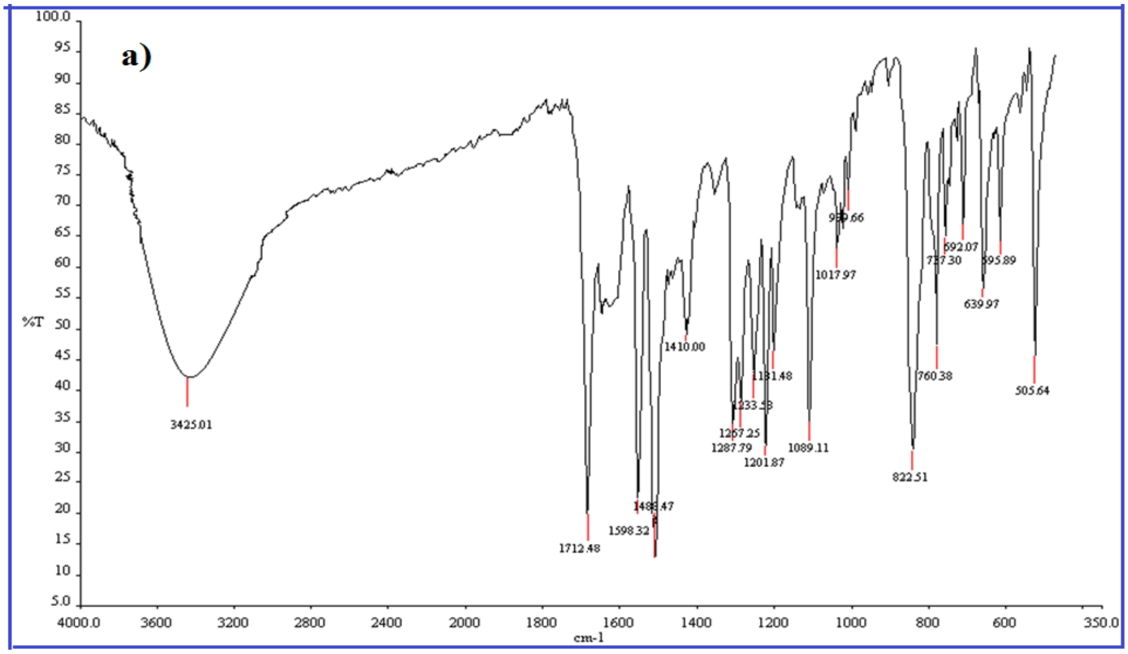

a) IR Spectra of Schiff base (DHPEAPMQ).

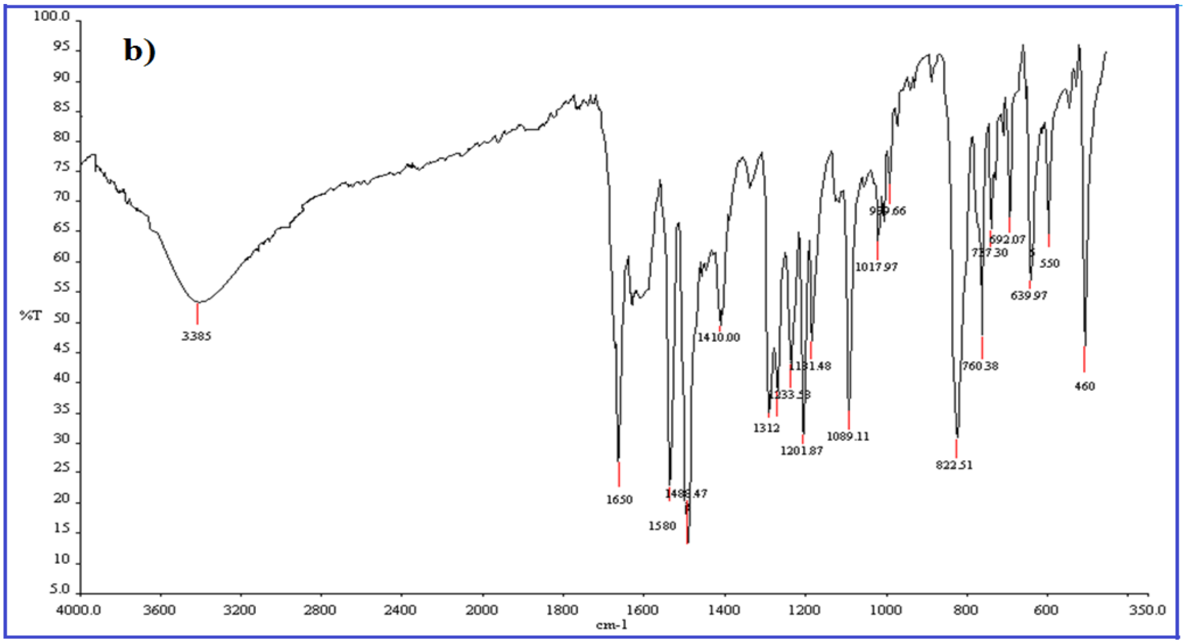

b) IR spectra of Cu(II) complex.

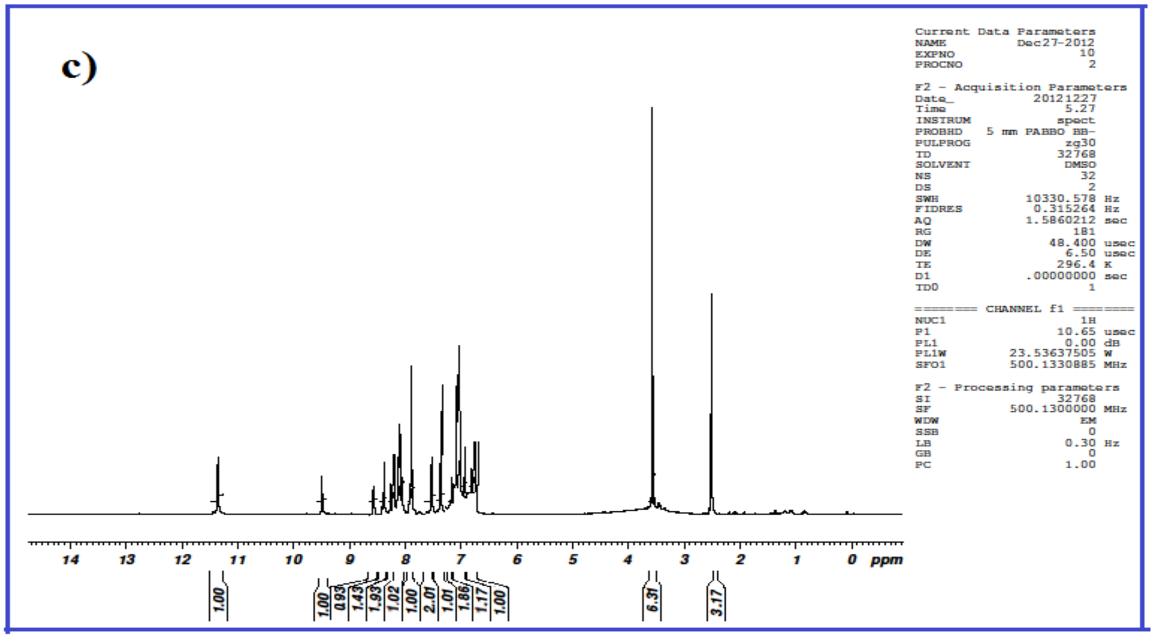

c) NMR spectra of Schiff base (DHPEAPMQ).

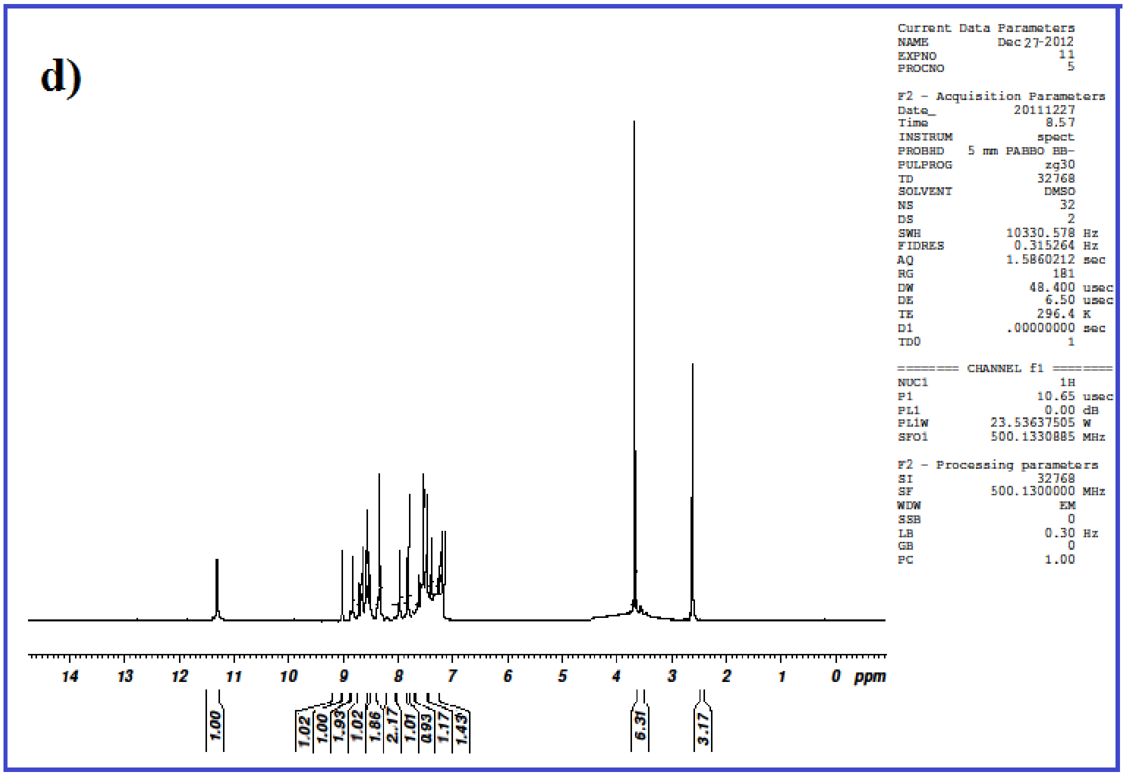

d) NMR spectra of Zn(II) complex.

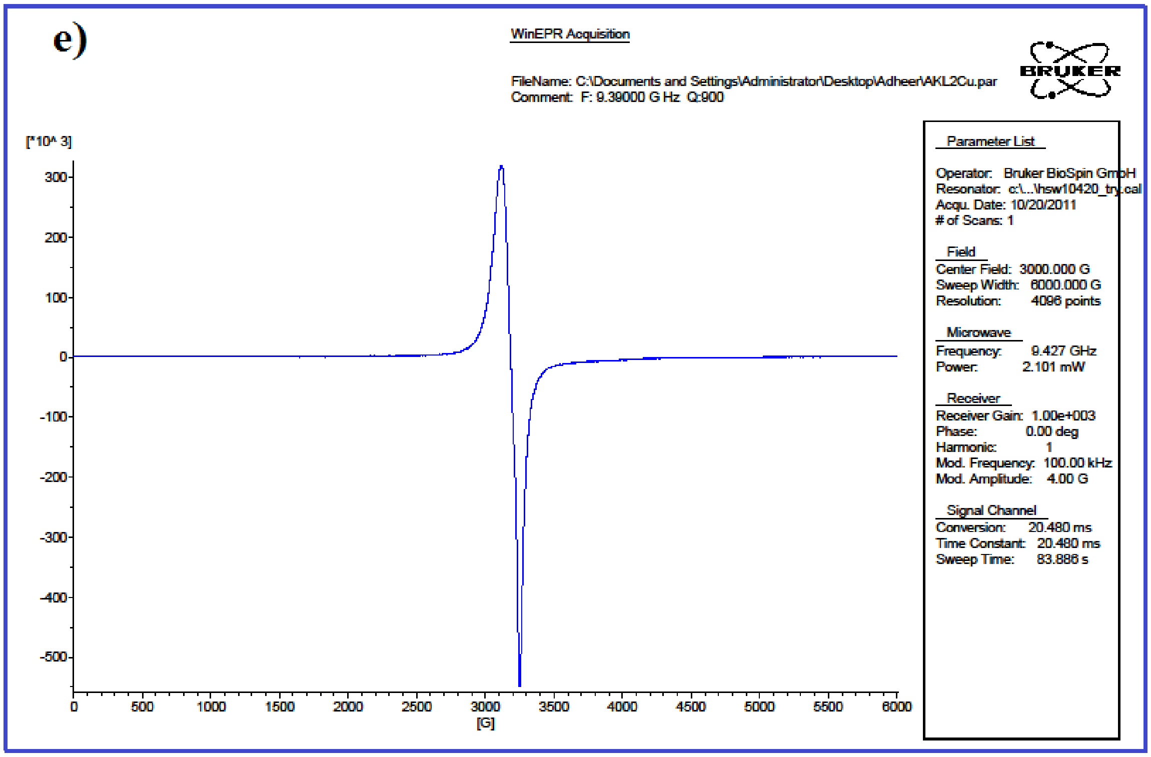

e) ESR spectra of Cu(II) complex.
